# Mechanics of Pickering Drops Probed by Electric Field–Induced Stress

**DOI:** 10.3390/ma10040436

**Published:** 2017-04-21

**Authors:** Alexander Mikkelsen, Paul Dommersnes, Zbigniew Rozynek, Azarmidokht Gholamipour-Shirazi, Marcio da Silveira Carvalho, Jon Otto Fossum

**Affiliations:** 1 Department of Physics, Norwegian University of Science and Technology, Høgskoleringen 5, NO-7491 Trondheim, Norway; almikkelsen87@gmail.com (A.M.); zbigniew.rozynek@gmail.com (Z.R.); jon.fossum@ntnu.no (J.O.F.); 2 Faculty of Physics, Adam Mickiewicz University, Umultowska 85, 61-614 Poznań, Poland; 3 Department of Mechanical Engineering, Pontifícia Universidade Católica do Rio de Janeiro, Rua Marquês de São Vicente, 225, Rio de Janeiro 22430-060, Brazil; a_gholamipour@esp.puc-rio.br (A.G.-S.); msc@puc-rio.br (M.d.S.C.); 4 Institut Pierre-Gilles de Gennes, 6-12 rue Jean Calvin, 75005 Paris, France

**Keywords:** Pickering drops, electric fields, drop deformation, drop retraction

## Abstract

Fluid drops coated with particles, so-called Pickering drops, play an important role in emulsion and capsule applications. In this context, knowledge of mechanical properties and stability of Pickering drops are essential. Here we prepare Pickering drops via electric field-driven self-assembly. We use direct current (DC) electric fields to induce mechanical stress on these drops, as a possible alternative to the use of, for example, fluid flow fields. Drop deformation is monitored as a function of the applied electric field strength. The deformation of pure silicone oil drops is enhanced when covered by insulating polyethylene (PE) particles, whereas drops covered by conductive clay particles can also change shape from oblate to prolate. We attribute these results to changes in the electric conductivity of the drop interface after adding particles, and have developed a fluid shell description to estimate the conductivity of Pickering particle layers that are assumed to be non-jammed and fluid-like. Retraction experiments in the absence of electric fields are also performed. Particle-covered drops retract slower than particle-free drops, caused by increased viscous dissipation due to the presence of the Pickering particle layer.

## 1. Introduction

Particles bound to drop interfaces by capillary forces can form a protective layer, preventing drop coalescence. This constitutes the basic mechanism for Pickering emulsion stabilisation [1,2,3]. Pickering layers can be either homogenous or heterogeneous in terms of particle compositions, and can be produced using microfluidic methods [3,4,5] or electric fields [6,7,8,9,10]. The particles at the drop interface can be locked (e.g., by sintering [11]) to form a rigid capsule, a colloidosome, which can have a wide range of applications, including molecular transport and release [12,13].

Controlled deformation of shells provides information about their mechanical properties [14]. External stresses can be applied by various means to study the mechanical and rheological properties of Pickering drops. Buckling has been measured by drop volume compression and expansion [15,16,17], and drop deformation and elasticity have been investigated by mechanical compression [18,19,20], hydrodynamic shear flow [21,22], and microfluidic focusing devices [23].

Electric fields have been used to manipulate particles on drop interfaces by utilizing dielectrophoretic forces [6,7,10,24] and electrohydrodynamic (EHD) flows [8,9,25,26,27,28,29,30]. In addition, Pickering drops may coalesce [9,31,32], and in some cases produce non-spherical-shaped jammed shells [9,33,34]. 

In the present work, we use external uniform DC electric fields to induce electric stresses to drops covered by particles. The electric stress can result in oblate deformation (compression) or prolate deformation (stretching), depending on the electric properties of the fluids and the particles. For example, for a capsule made out of silicone oil covered by polyethylene (PE) particles and suspended in castor oil, the electric stress induces a compressive deformation along the applied field direction. The deformation can be controlled by varying the strength of the applied electric field. To prepare these drops that are fully covered by granular or colloidal particles, we use an electric field-driven self-assembly route, as described in detail in our previous work [8,9,35].

## 2. Results

### 2.1. Electric Field–Induced Deformation of Pickering Drops

**Figure 1:**
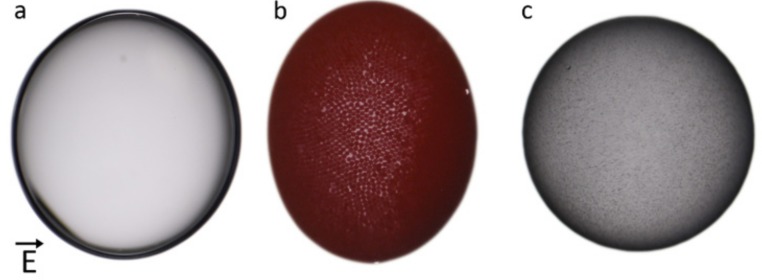
Electric field–induced deformation of pure and particle-covered silicone oil drops in castor oil. (**a**) Pure drop ( D=−0.04 ), (**b**) PE particle-covered drop (D=−0.13), and (**c**) clay particle-covered drop (D=−0.02) subjected to the same electric field strength, *E* = 250 Vmm^−1^. The initial diameter before deformation of each drop was 1.2 mm. The electric field direction is horizontal (as indicated by the arrow).

Pickering drops are here prepared by adding particles to silicone oil, and by immersing the silicone oil dispersion in castor oil. The particle concentration in the silicone oil is tuned such that the resulting Pickering particle layer is not jammed, and in equilibrium the Pickering drops are spherical in the absence of electric fields (for more details, see Materials and Methods section). In Figure 1, the electric field-induced deformation of a pure silicone oil drop is compared with the deformation of a silicone oil drop covered by PE particles (estimated particle coverage 84% ± 2%) and a silicone oil drop covered by clay particles (estimated particle coverage 81% ± 7%, see Materials and Methods for details regarding the protocol for the estimation of particle coverage). At this field strength (250 Vmm^−1^), the drop covered with PE particles (Figure 1b) is more deformed than the pure drop (Figure 1a), while the drop covered by clay particles (Figure 1c) is less deformed than the pure silicone oil drop.

**Figure 2:**
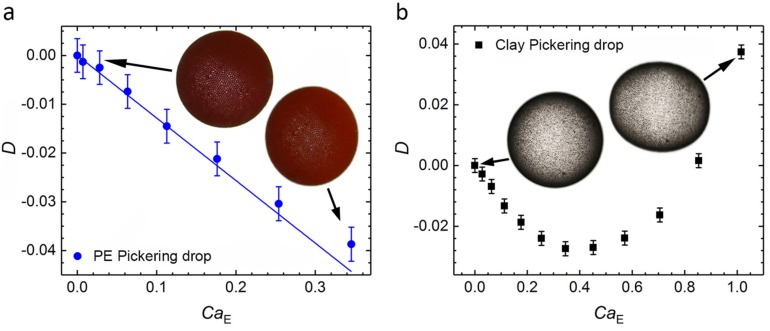
Pickering drop deformation. (**a**) Pickering drop made of silicone oil and PE particles suspended in castor oil. Subjected to a uniform DC electric field (horizontal in the inserted pictures), the Pickering drop deforms into an oblate geometry. The Pickering drop deformation (*D*) is plotted versus the electric capillary number $Ca_{E}=\varepsilon_{0}\varepsilon_{\mathrm{ex}}E_{0}^{2}r_{0}/\gamma$. The experimental data is fitted with a fluid shell description (discussed in the Discussion section and described in details in Supplementary Materials). (**b**) A Pickering drop made out of silicone oil and Li-Fh clay particles (see the Materials and Methods section for the description of clay particles) suspended in castor oil. When subjected to weak electric fields, this drop deforms into an oblate shape. As the electric field increases, the drop deforms to a spherical shape and then becomes prolate when $Ca_{E}$ > 0.85 at stronger fields. The initial radius before deformation of each drop was 1.2 mm.

The drop deformation is defined as $D=(d_{\left|\right|}-d_{⊥})/\left(d_{\left|\right|}+d_{⊥}\right)$, where $d_{\left|\right|}$ and $d_{⊥}$ are the drop axes parallel and perpendicular to the electric field direction, respectively. The electric field–induced deformation of the two Pickering drops above is further investigated and presented in Figure 2, which displays the deformations of the Pickering drops plotted versus the dimensionless electric capillary number. The electric capillary number is defined as: $Ca_{E}=\varepsilon_{0}\varepsilon_{\mathrm{ex}}E_{0}^{2}r_{0}/\gamma$, where $\varepsilon_{0}$ is the vacuum permittivity, $\varepsilon_{\mathrm{ex}}$ is the dielectric constant for the exterior fluid, $E_{0}$ is the applied electric field, γ is the drop surface tension and $r_{0}$ is the drop radius. As noted above, for insulating PE particles, we observe enhanced oblate deformation compared with pure silicone oil drops (Figure 2a). In Figure S1, the deformation of pure silicone oil drops and PE Pickering drops with different diameters ranging from 1.2 to 2.2 mm are plotted versus the electric capillary number. The PE Pickering drops consistently deform more than pure silicone oil drops of similar size, when subjected to the same electric field strength. The clay Pickering drops behave differently (Figure 2b); for weak electric fields, the deformation is oblate and a maximum negative deformation is observed around $Ca_{E}$ = 0.35. As the electric field increases, the drop deforms to a spherical shape and becomes prolate (positive deformation) when $Ca_{E}$ > 0.85.

Because the conductivity of PE is ohmic, the experimental data in Figure 2a can be fitted (blue line) to the fluid shell description (see details in Supplementary Materials and Figure S2) to obtain a value of the electrical conductivity of the Pickering film, $\sigma_{f}$. The conductivity of clay particles is non-ohmic, and the relationship between the electric current and electric voltage is unknown for the clay Pickering film. Thus, to find this relationship, the conductivity of the clay film has to be calculated for each data point in Figure 2b and then fitted by a function, as discussed in detail in section 3.1.

### 2.2. Surface Tension of Pickering Drops in the Absence of Electric Fields

**Figure 3:**
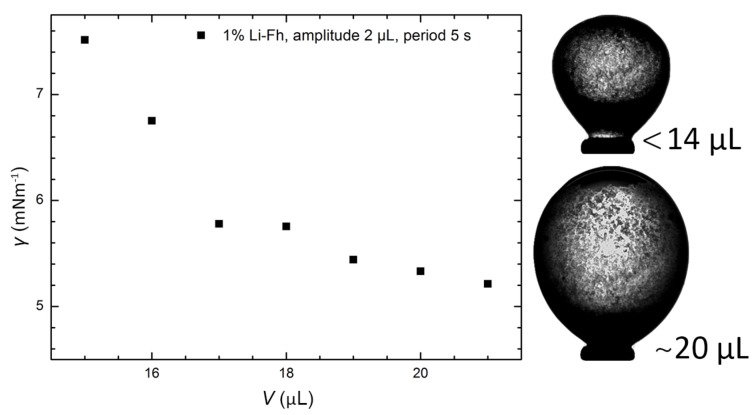
Surface tension of Li-Fh clay Pickering drops. Measured surface tension (γ) plotted versus drop volume (V) for Pickering drops made out of 10 cSt silicone oil, suspended in castor oil and covered with Li-Fh clay particles. The bottom right picture is of a clay-covered Pickering drop where the particle layer is fluid (volume around 20 µL), which crumples as the volume decreases below 14 µL (top right picture). The silicone drop volume is controlled by a syringe. The surface area decreases with the volume as $A=CV^{2/3}$, and the particle layer goes from fluid (where the surface particles have enough space to move) to an elastic solid as the particle packing increases and the particles start to jam. For the largest Pickering drop volume, the measured surface tension of the Pickering drop is approximately the surface tension between silicone and castor oil (4.5 mNm^−1^).

The observed change in deformation of Pickering drops compared to particle-free drops can be caused by a change in electrical stress and/or by a change in resistance to deformation related to surface tension. It is therefore important to know how the surface tension changes when particles are added to the drop interface. Oscillating drop tensiometry is here used to estimate the surface tension of clay and PE Pickering drops in the absence of electric fields. Figure 3 displays clay-covered Pickering drops where the packing is low (large volume) and high (small volume). The measured surface tension between the Pickering drop particle layer and the surrounding castor oil increases with the particle packing (inversely proportional to the drop volume). We also measured the surface tensions to be 4.6 ± 0.2 mNm^−1^ for silicone oil drops in castor oil, and 5.0 ± 0.5 mNm^−1^ for PE Pickering drops in castor oil.

## 3. Discussion

### 3.1. Pickering Drop Deformation in an Electric Field

If a drop is less conducting than the surrounding fluid, the drop’s overall dipole moment can be oriented anti-parallel with the electric field direction [36], and the electric stress distribution can induce oblate drop deformation (compressed in the direction of the electric field). More precisely, drops are oblately deformed if the Maxwell charge relaxation time [36] of the drop is longer than that of the surrounding fluid, and they become prolate (elongated in the direction of the electric field) in the opposite case, as shown by Taylor [37]. The observed oblate deformation of a pure silicone oil drop (Figure 1a) is consistent with the charge relaxation times calculated from the fluid parameters listed in Table 1 in the Materials and Methods section.

The observed increase (~300%) in steady-state deformation of PE Pickering drops compared to particle-free drops can in general be caused by: (i) reduced resistance to deformation related to reduced surface tension, (ii) added elastic stress from the particle shell, or (iii) an increase in electrical stress.

Because the measured change in surface tension between the PE Pickering drop and the particle-free drop is much smaller (~10%, see Section 2.1) than the change in deformation (Figure 1), the increased PE Pickering drop deformation cannot be caused by a reduced surface tension (since the deformation of leaky electric drops is inversely proportional to the surface tension [37]). Particle-particle capillary interactions can induce resistance to two-dimensional shear deformation of the Pickering layer [38]. However, such elastic stress will only cause resistance to deformation, and should therefore not be the cause of the increased deformation of the PE Pickering drops. We also performed experiments in an alternating current (AC) electric field (Figure S3). In this case, there is no free charge build-up at drop interfaces, which means that only dielectric forces are involved in the deformation, and the PE Pickering drop is slightly less deformed than the pure silicone oil drop. In the absence of free charge build-up, the electric stress on the drop interface is expected to be approximately the same for a pure drop and a Pickering drop, because the dielectric constant of PE is very similar to the dielectric constant of silicone oil, and because the volume of the PE particle layer is small compared to the volume of silicone oil. This result supports the assumption that the resistance to deformation (due to Laplace pressure and/or elastic modulus of the Pickering shell) is approximately the same for a pure drop and a PE Pickering drop, provided that the particle layer is below the granular jamming transition, as is the case in Figure 1 and Figure 2.

Because both (i) and (ii) are ruled out as the main cause, the increase in deformation of the PE Pickering drop is expected to be caused by increased electric stress that results from larger charge accumulation at the drop interface. The increased charge build-up can be due to both a decrease in electric conductivity of the drop interface and absence of electrohydrodynamic charge convection [39]. The strength of an electrohydrodynamic charge convection mechanism is quantified by the electric Reynolds number $Re_{E}$, which is defined as the ratio of the Maxwell-Wagner charge relaxation time $\tau_{e}$ (time for charge to build up at the interface [36]) to the flow charge convection time $\tau_{f}$ (time for charge transport by convection [36]). For the silicone oil drop in castor oil system, $\tau_{e}$ is of the same order as $\tau_{f}$. Thus, $Re_{E}$ ≈ 1, and charge convection may be significant for pure drops [39]. This is not the case for Pickering drops, because the tangential electric stress is absorbed in the particle layer, and electrohydrodynamic circulation flows (viscous forces) are supressed [8,9,28] (in accordance with our observations) allowing for larger charge accumulation.

When silicone oil drops are covered by clay particles instead of PE particles, the conductivity of the drop interface increases in comparison to that of both the PE Pickering drop and pure silicone oil drop. As a result, the clay Pickering drops behave differently with an oblate-prolate deformation transition (Figure 2b). In addition, the conductivity of clay particle dispersions is non-ohmic and increases with the applied electric field [40]. Thus, the conductivity of the clay Pickering film is also expected to increase with the electric field.

Electric field-induced deformation of leaky-dielectric drops has been extensively studied and modelled [36,37,39,41]. Theoretical models have also been developed for vesicles and membrane-covered drops [42,43], and recently also for particle encapsulated drops [28,44,45]. These works consider an elastic particle layer on the capsule, i.e., a particle layer with shear elasticity.

In our present experiments on PE particle-covered drops, the surface concentration is sufficiently low for the particle network to restructure upon deformation, i.e., the particle layer can be considered to be fluid. We have previously shown experimentally that our clay Pickering films are easily deformed and fluid-like [8]. Therefore, here we describe the Pickering drop layer as thin fluid shell. With this assumption we find that the expression for the electric field induced deformation is:

$D\approx \frac{E_{0}^{2}\varepsilon_{0}}{\gamma_{0}\sigma_{f}{\left(2\sigma_{\mathrm{ex}}+\sigma_{\mathrm{in}}\right)}^{3}}(\alpha_{0}+\alpha_{1}d).$ The α coefficients are functions of the dielectric constants, conductivities of the materials and drop radius (see Supplementary Materials for details on the calculation). The fluid shell thickness is d, and its electric conductivity and dielectric constant are $\sigma_{f}$ and $\varepsilon_{f}$, respectively. The electric conductivity and dielectric constant of the silicone oil drop are $\sigma_{\mathrm{in}}$ and $\varepsilon_{\mathrm{in}}$, and those of the exterior liquid (castor oil) are $\sigma_{\mathrm{ex}}$ and $\varepsilon_{\mathrm{ex}}$. The electric Maxwell stress working on a leaky-dielectric drop has normal and tangential components. The normal component of the electric stress is balanced by the Laplace pressure with a non-uniform surface tension $\gamma =\gamma_{0}+\delta \gamma \cos2\theta$, where θ is the polar angle, $\gamma_{0}$ is the uniform component of the surface tension and δγ is determined from the tangential stress balance [46]. In our description, the tangential component of the electric stress is balanced by the gradient of the surface tension of the Pickering film ∇γ, which is due to particle-particle interactions. For PE particles these may be capillary-mediated interactions [38], whereas clay particles may also be cohesive. The dielectric constants of the fluids and particles, the electrical conductivities of the fluids and the interfacial surface tensions are estimated from independent measurements and are given in the Materials and Methods section. Using the fluid shell description and the data points in Figure 2a, we estimate the electric conductivity of the PE Pickering film to be ~30% of the electrical conductivity of the silicone oil, which is much larger than the conductivity of PE. The conductivity of PE is approximately seven orders of magnitudes smaller than that of the silicone oil (see Materials and Methods). However, because the PE particle layer on the Pickering drop is porous (we observe that there is silicone oil between the PE particles), the conductivity of the Pickering PE layer is expected to be closer to that of silicone oil than to the conductivity of PE.

**Figure 4:**
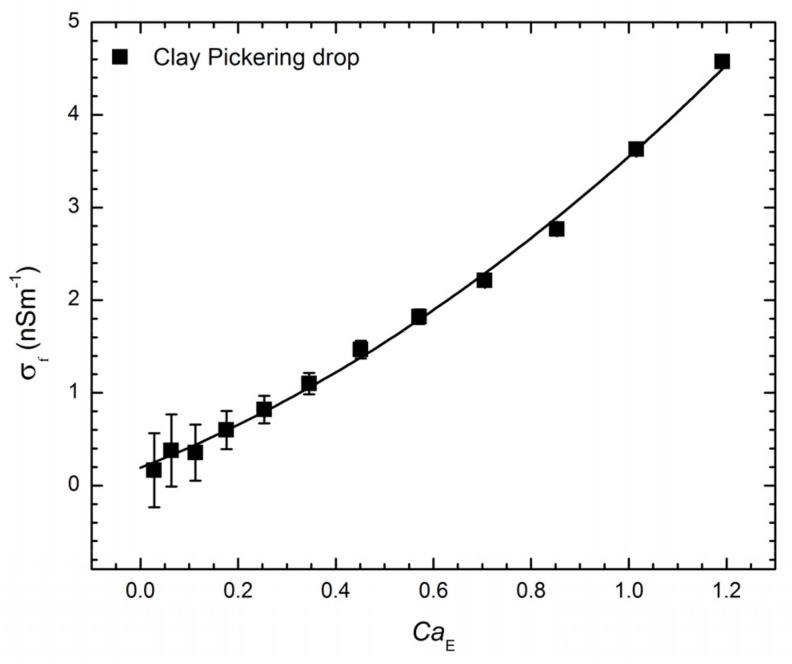
Estimated electric conductivity of Li-Fh clay particle film. The data points are a plot of the estimated electrical conductivity$\left(\sigma_{f}\right)$ of the clay Pickering layer as a function of the electric capillary number $\left(Ca_{E}\right)$, calculated from the fluid shell description (see details in Supplementary Materials) using the deformation data in Figure 2b. The full line is a polynomial fit, as described in the text.

Due to the non-ohmic response of clay particles (reported previously in [40]), the electric field dependence on the conductivity of the Pickering particle layer is unknown. For this reason, the fluid shell description cannot be used to analytically calculate an expression for conductivity of the particle layer from the drop deformation (Figure 2b). Instead, we first calculate the conductivity of the clay Pickering film numerically from the measured deformation data in Figure 2b at different electric capillary numbers (proportional to $E_{0}^{2}$). The calculated values of conductivities are shown in Figure 4. We then fit the data points in Figure 4 with a polynomial function, which gives the particle film conductivity as a function of the applied electric field: $\sigma_{f}\approx 1.98\times 10^{-10}\;{\mathrm{Sm}}^{-1}+2.30\times 10^{-20}E_{0}^{2}\;{\mathrm{SmV}}^{-2}+1.67\times 10^{-31}E_{0}^{4}\;{\mathrm{Sm}}^{3}V^{-4}.$ The electric permittivity of clay can also change with electric field. However, the change in the electric permittivity of clay is reported to be much smaller than the change in electric conductivity [40]. 

### 3.2. Relaxation of Drop or Pickering Drop Deformation after Turning off the Electric Field

**Figure 5:**
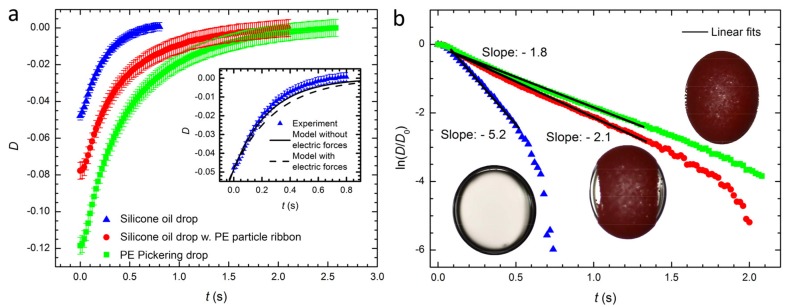
Drop relaxation. Retraction of a silicone oil drop without particles (▲), a silicone oil drop covered by surface PE particles (estimated particle coverage 75% ± 3%) forming a ribbon (●) and a PE Pickering drop (estimated particle coverage 84% ± 2%) (■). The applied DC electric field (250 V/mm) is turned off when the time *t* = 0. The inset in the figure (**a**) compares the experimentally measured relaxation of a pure silicone oil drop with two theoretical models for drop relaxation: (i) only considers capillary forces [48] and (ii) considers both capillary and electric forces [49]. (**b**) $\ln\;\left(D/D_{0}\right)$ plotted versus time for the drops in (**a**). Here $D_{0}$ is the steady-state deformation before the electric field is turned off. The inset pictures in (**b**) show the drops used for the relaxation experiments before the electric field was switched off. The PE particle size is 47–52 μm, and the applied electric field direction is horizontal in the inserted figures in (**b**). The initial radius before deformation of each drop was around 1.2 mm.

In the present experiments, all Pickering and pure drops relax to a spherical shape when the electric field is turned off. Figure 5a shows the transient relaxation dynamics of a pure silicone oil drop (blue triangles), a silicone oil drop with PE particle ribbon (red circles), and a PE Pickering drop (green squares). In Figure 5b it is evident that the silicone oil drop relaxes to a spherical shape (*D* = 0) faster than silicone oil drops covered with PE particles, and that in all cases the relaxation is exponential $D\left(t\right)=D_{0}e^{-\frac{t}{\tau }}$, where $D_{0}$ is the initial deformation, *t* is the time elapsed since the electric field is removed and τ is the relaxation time. The experimental relaxation times of the three cases: $\tau_{\mathrm{silicone}}$ = 0.19 s for a pure silicone oil drop, $\tau_{\mathrm{ribbon}}$ = 0.48 s for a silicone oil drop covered with a PE particle ribbon, and $\tau_{\mathrm{Pickering}}$ = 0.56 s for a PE Pickering drop. The characteristic relaxation time for a silicone oil drop covered by PE particles (estimated particle coverage around 75% and 84%, see Materials and Methods) is significantly longer than that of a pure silicone oil drop.

For the case of a pure drop, balancing capillary and viscous forces [47,48] gives the relaxation time $\tau =\tau_{d}=\left[\mu_{\mathrm{ex}}r_{0}\left(2\lambda +3\right)\left(19\lambda +16\right)\right]/\left[\gamma \left(\lambda +1\right)\right]$. Here γ is the drop surface tension, $r_{0}$ is the radius of the drop, $\mu_{\mathrm{ex}}$ is the viscosity of the exterior liquid and λ is the ratio of the drop viscosity and the exterior fluid viscosity. Inserting numbers in the equation above, the theoretical relaxation time for a pure silicone oil drop with a size of 2.4 mm is $\tau_{\mathrm{silicone}}$ = 0.25 s, which is close to our experimental observation given above. Even though the electric field is turned off, electric forces can still contribute to the relaxation dynamics of pure drops, because the free charges accumulated at the drop interface discharge with the finite Maxwell-Wagner charge relaxation time, which is $\tau_{MW}$ ≈ 1 s for this system [8]. These free charges interact and can create a force that can influence the shape-relaxation [49]. The inset in Figure 5a compares the experimentally measured relaxation of a pure silicone oil drop with the two theoretical models for drop relaxation: (i) only considers capillary forces [48] and (ii) considers both capillary and electric forces [49]. The comparison shows that the effect of electric forces after the electric field is turned off can be neglected here, and we will therefore use the model that only consider capillary driven relaxation.

Surface-adsorbed particles can in some cases reduce the Laplace pressure of the interface [16], which can also slow down the relaxation [50]. However, we do not observe any significant difference in the surface tension of a particle-free silicone oil drop in castor oil (4.5 mNm^−1^) compared with a PE Pickering silicone drop in castor oil (5.0 mNm^−1^). The changes in the relaxation time between these drops should therefore be due to viscous dissipation caused by the presence of the particle layer. The PE particle layer imposes constraints on the hydrodynamic flow inside and outside the drop, which can increase the viscous dissipation. In addition, the fluid PE particle layer may in itself possess an effective two-dimensional viscosity due to hydrodynamically mediated friction between particles as they glide along each other [35].

## 4. Conclusions

We have shown that the deformation of pure and particle-laden drops can be controlled by varying the strength of the applied electric field and the drop size, and that the deformation magnitude and sign depends on the electrical conductivity of the drop interface. The electrical conductivity of the drop interface changes when adding surface particles and can be tuned by both the particle coverage and electrical properties of particles. We have developed a fluid shell description for the steady-state deformation of Pickering drops covered by a particle layer that is assumed to be non-jammed and fluid-like. This is used to estimate the conductivity of insulating PE and conductive clay particle layers on Pickering drops. Retraction experiments in the absence of electric fields demonstrate that particle-covered drops retract more slowly than particle-free drops. This is mainly caused by increased viscous dissipation due to the presence of the Pickering particle layer.

We have shown how mechanical and electric properties of Pickering drops with different properties can be probed by electric fields. The method could be further developed into a tool for characterizing Pickering films and to study rheological properties of Pickering drops. The observed rheological response could be similar to the dynamics of Pickering drops in hydrodynamic shear flow, which can deform a drop and cause unjamming of the Pickering layer.

## 5. Materials and Methods

### 5.1. Materials

The Pickering drops used in our experiments were made out of 50 cSt silicone oil (Dow Corning 200/50 cSt, electric conductivity $\sim 5.6\;{\mathrm{pSm}}^{-1}$, relative permittivity 2.8 and density $\sim 0.96\;{\mathrm{gcm}}^{-3}$) and polyethylene particles (purchased from Cospheric LLC with electric conductivity $<1\times 10^{-20}\;{\mathrm{Sm}}^{-1}$, relative permittivity ~2.1, density $\sim 1.0\;{\mathrm{gcm}}^{-3}$, and diameter ~50 μm) or clay particles (Li-fluorohectorite, a synthetic 2:1 clay, comes from the same batch of materials as reported and characterised by Hansen et al. [51] and references therein, polydispersed in size, from hundreds of nm to 10 µm, electric conductivity ~90 μSm^−1^ [8] and dielectric constant ~6). The contact angle of PE particles at the interface of a silicone oil drop is measured to 60° ± 3° (against castor oil), which indicates that the PE particles have slightly higher affinity towards silicone oil. The silicone oil and particles were measured by weight, stirred together, shaken and ultrasonicated to minimise particle aggregation. Castor oil (Sigma-Aldrich 83912, specific density of $\sim 0.96\;{\mathrm{gcm}}^{-3}$, electric conductivity $\sim 56\;{\mathrm{pSm}}^{-1}$, relative permittivity ~4.7 and viscosity ~750 cSt) was poured in a sample cell (15 × 15 × 30 mm) consisting of glass and two indium tin oxide (ITO) walls, constituting electrodes. The silicone oil mixture was immersed in castor oil using a regular mechanical pipette.

### 5.2. Estimation of Particle Coverage

To quantify the coverage of particle at drop surfaces, images were computer processed. The particle coverages were calculated to be 75% ± 3% for a drop with PE particles forming a ribbon (inset image in Figure 5b), and 84% ± 2% for a PE covered drop (Figure 1b and the inset image in Figure 5b). We also attempted to estimate the clay particle coverage on the drop shown in Figure 1c. However, the clay particles are much more polydispersed in both size and shape, and can also form a multilayered film. It was therefore challenging to distinguish voids in particle film from the parts of the film that is very thin and transparent. The particle concentration was estimated to be 81% ± 7%, but the value can be even closer to 100%.

### 5.3. Electrical Measurements

Pictures were recorded using a digital camera mounted on a stereoscope, and the observation view was always perpendicular to the electric field, which in the figures are in the horizontal direction. Particle sedimentation was used to bring particles to the drop interfaces where they adsorbed to the oil-oil interface due to capillary binding forces [2,52]. With particles confined on the drop surfaces, the application of an electric field across the sample cell induces EHD liquid flows [36,37] inside and outside the drop that are utilised to self-assembly granular (PE) and colloidal (Li-Fh) particles on the drop surface and eventually form Pickering drops. Extensive details of the method can be found in our previous work [8]. 

### 5.4. Oscillation Drop Tensiometry

We used oscillating drop tensiometry to measure the interfacial rheology of Pickering drops. Silicone oil (Dow Corning 200/10 cSt, viscosity of 10 cSt, electric conductivity $\sim 5.6\;{\mathrm{pSm}}^{-1}$, relative permittivity ~2.8 and density $\sim 0.93\;{\mathrm{gcm}}^{-3}$) with clay particles was used as the drop phase, while castor oil was used as the external phase. The clay particles were crushed and only the smallest particles <10 µm were selected by centrifugation. The density of the silicone oil and clay solution was measured to be 0.931 gcm^−3^ for silicone oil with 1% Li-Fh. The silicone oil–clay solution was extracted to a 500 µL gas-tight syringe and mounted to the equipment, while the castor oil was poured into a glass cuvette. The light silicone oil–clay drop was then made upward at the tip of a J-bent steel needle (outer diameter: 1.2 mm) and immersed in the castor oil. The tensiometer (Tracker S, Teclis instruments) uses a piezoelectric actuator to control the drop volume and an axisymmetric drop profile analysis technique. After the drop was made by injecting fluid, an external DC electric field (250 Vmm^−1^) was applied for 10 min to bring and structure the clay particles at the drop interface. With all the clay particles adsorbed at the interface, we used WDROP (version T2011) software to apply a sinusoidal variation of the drop volume while measuring the Pickering drop shape. Starting with a drop volume of 15 µL, the amplitude was set to 2 µL and the period to 5 s. All experiments were performed at 23 °C and were run for at least 5 min. The interfacial surface tension and elasticity were then measured using WDROP, which analysed the drop shape and fit the shape to the Young-Laplace equation. Depending on the apparatus, neglecting viscous forces is considered safe when the capillary number (the ratio between viscous and capillary forces) Ca<0.002 [53]. For the surface tension experiments conducted here, Ca is around 0.06, which means that viscous forces are too strong to be neglected and that elasticity values obtained from this method are found to be inaccurate. Also note that at high oscillation rates (compared to the inverse of the capillary time scale), hydrodynamic effects can lower the accuracy of dynamic surface tension measurements [54,55].

## Figures and Tables

**Table 1:** Summary of material and estimated system parameters. Set of parameters for a silicone oil drop suspended in castor oil. The top section of the table lists material parameters for the drop and medium, while the bottom section lists dimensionless numbers for the system, as defined in Supplementary Materials. For the estimates of the electrical capillary number $Ca_{E}$ and the electrical Reynolds number $Re_{E}$, an electric field strength of 200 Vmm^−1^ is used. The other dimensionless numbers listed: the fluid-fluid dielectric ratio S, viscosity ratio λ, and electrical conductivity ratio R, γ is the surface tension between silicone oil and castor oil. These quantities are all independent of the electric field strength.

Fluid	$\varepsilon_{r}$	σ (Sm^−1^)	μ (Pa s)	ρ (kgm^−3^)	$r_{0}$ (mm)
Drop (silicone oil)	2.8	$5.6\times 10^{-12}$	0.05	961	1.0
Medium (castor oil)	4.7	$5.6\times 10^{-11}$	0.75	960	-
$Ca_{E}$	$Re_{E}$	S	λ	R	γ **(mNm^−1^)**
0.4	1.3	1.7	0.07	0.1	4.5

## Supplementary Materials

The following are available online at www.mdpi.com/1996-1944/10/4/346, Figure S1: Deformation of particle free silicone oil drops and PE Pickering drops plotted versus the electrical capillary number, Figure S2: Geometry of a dielectric spheroid capsule subjected to a uniform DC electric field, Figure S3: Deformation of a particle free silicone oil drop and PE Pickering drop subjected to AC electric field.
