# Supplementary material for: Mechanics of Pickering Drops Probed by Electric Field–Induced Stress
Source: Materials (Basel). 2017 Apr 21;10(4):436. doi: 10.3390/ma10040436 (PMC5506933; doi:10.3390/ma10040436)
Supplement: Supplementary file 1 [file materials-10-00436-s001.pdf]

Supplementary Materials: Mechanics of Pickering Drops Probed by Electric Field–Induced Stress

Alexander Mikkelsen, Paul Dommersnes, Zbigniew Rozynek, Azarmidokht Gholamipour-Shirazi, Marcio da Silveira Carvalho and Jon Otto Fossum

Deformation of Drops with Different Sizes Subjected to a Range of Direct Current (DC) Field Strengths

The electric capillary number $Ca_{\mathrm{E}} = \varepsilon_0 \varepsilon_{\mathrm{ex}} E_0^2\, r_0/\gamma$ is a measure of the strength of the electric force versus capillary forces. ε_0 is the vacuum permittivity, $\varepsilon_{\mathrm{ex}}$ is the dielectric constant for the exterior fluid, E_0 is the applied electric field, γ is the drop surface tension, and r_0 is the drop radius. Figure S1 displays drop deformation for both silicone oil and Pickering drops with different sizes plotted versus Ca_{E}.

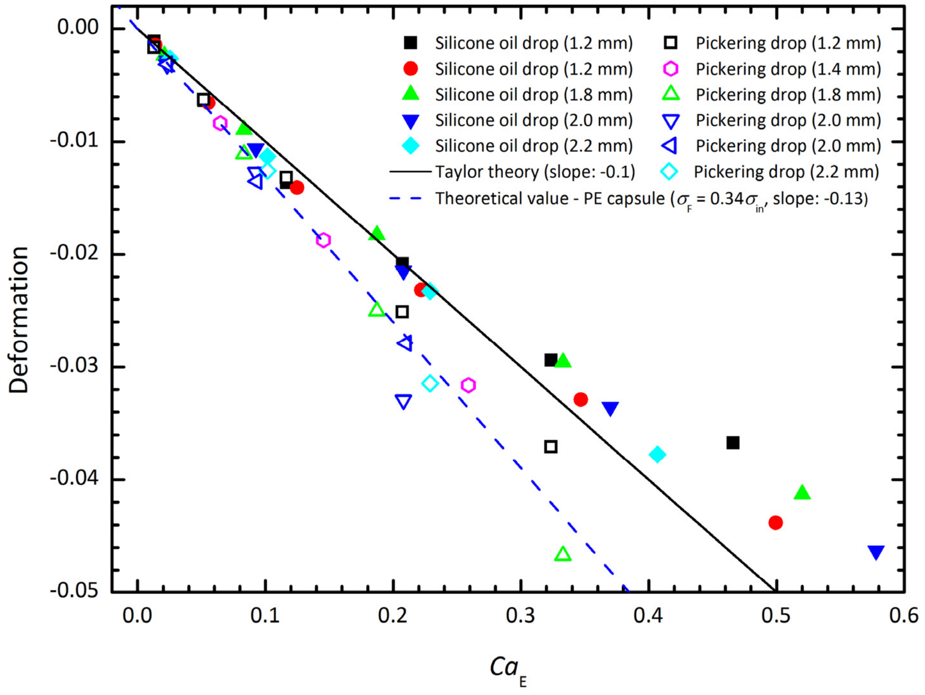

Figure S1. Deformation of particle-free silicone oil drops and polyethylene (PE) Pickering drops plotted versus the electrical capillary number Ca_{E}. The Pickering drops are silicone oil drops covered by 47–52 μm PE particles, and the drop size ranges between 1.2 and 2.2 mm. The solid line is Taylor's theory for linear drop deformation calculated using the parameters listed in the Methods section. The dashed line is our fluid shell description, using a Pickering PE surface conductivity that is 30% of the silicone oil conductivity.

Classical Taylor Electrohydrodynamic (EHD) Theory for Emulsions Drops

EHD deformation of conducting emulsion drops, free of particles, is described by the Taylor leaky dielectric model [1], which has been developed by Melcher [2] and others [3–5]. Free charge build-up at the drop interface creates interfacial electrical stresses that are balanced by the drop surface Laplace pressure and viscous hydrodynamic stresses. The Taylor model gives the drop deformation: $D = (d_{\parallel} - d_{\perp})/(d_{\parallel} + d_{\perp}) = \frac{9 r_0 \varepsilon_0 \varepsilon_{\mathrm{ex}} E_0^2}{16 \gamma S (2+R)^2}\left[S(R^2+1) - 2 + 3(RS-1)\frac{2\lambda+3}{5\lambda+5}\right]$, where $d_{\parallel}$ and $d_{\perp}$ are the drop axis lengths parallel and perpendicular to the electric field direction, respectively, $\varepsilon_{\mathrm{ex}}$ is the dielectric constant of the surrounding fluid, r_0 is the drop radius, γ is the interfacial surface tension between the drop and exterior fluid, and the dimensionless numbers R, S and λ are the

conductivity, dielectric constant and viscosity ratios: $R = \sigma_{\mathrm{in}}/\sigma_{\mathrm{ex}}, S = \varepsilon_{\mathrm{ex}}/\varepsilon_{\mathrm{in}}, \lambda = \mu_{\mathrm{in}}/\mu_{\mathrm{ex}}$, where σ is electric conductivity, ε is dielectric constant, and μ is viscosity. The subscript "ex" refers to the exterior surrounding liquid (castor oil) while "in" refers to the interior drop (silicone oil). The drop deformation can be oblate ($D < 0$) or prolate ($D > 0$), depending on these parameters. Notice that the Taylor model is only valid for weak drop deformations and when the charge relaxation time is much shorter than the charge convection time.

Electric Field-Induced Deformation of a Pickering Drop: Fluid Shell Description Developed for the Current Work

When subjected to an electric field, electric stress is induced at the surface of Pickering drops. If the drop is fully covered with particles, there is no EHD circulation flow in steady state because the particle layer adsorbs the tangential stress. Ouriemi and Vlahovska [6] modelled Pickering drops with the assumption that the electric stress is adsorbed by in-plane elastic deformation, i.e., the Pickering layer is considered as an elastic capsule. In our experiments, we observed that clay-covered Pickering drops appear to be more fluid or gel-like for moderate particle coverage, and can undergo considerable in-plane deformation. For polyethylene (PE) bead-covered drops that are not jammed, we had previously observed that the beads can roll over each other in glide planes [7], which relaxes the in-plane elastic stress, giving a more fluid-like behaviour of the Pickering layer. Therefore, we here consider a fluid membrane description for the Pickering drop as a first approximation, where the Pickering drop layer is considered to be a uniform thin film (fluid membrane) of thickness d, with electrical conductivity and dielectric constant of σ_{F} and ε_{F}, respectively.

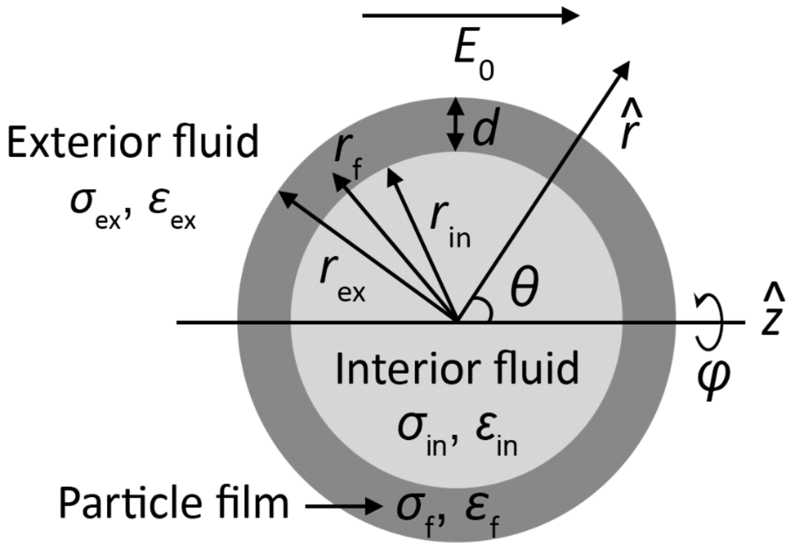

Figure S2. Geometry of a dielectric spheroid capsule subjected to a uniform DC electric field. The capsule is considered to be a sphere of an interior fluid coved by a particle film and surrounded by an exterior fluid. The dielectric constants ε and conductivities σ are marked in the figure and denoted "f", "ex", and "in", referring to the particle film, exterior and interior fluids, respectively. A spherical coordinate system is used where θ is the inclination angle, φ the azimuthal angle and r the distance from the centre of the capsule. The $r_{\mathrm{ex}}, r_{\mathrm{in}}$ parameters are the radii of the two interfaces, and d is the thickness of the particle film.

Here we consider a leaky-dielectric (weakly conductive) drop suspended in a leaky-dielectric exterior fluid and covered by a dielectric particle film with thickness $d = r_{\mathrm{ex}} - r_{\mathrm{in}}$. The capsule is subjected to a uniform DC electric field E_0 in the $\hat{z}$ direction $\vec{E} = E_0\hat{z}$ (Figure S2).

To calculate the electric field at the three different regions (capsule exterior, film and interior), we solve Laplace's equation $V_i(r,\theta) = (A_i r + B_i/r^2)\cos\theta$ with the usual boundary conditions for leaky dielectrics:

$$\lim_{r\to\infty} E_{\mathrm{ex}} \to E_0, \quad (1)$$

$$\lim_{r\to 0} |E_{\mathrm{in}}| < \infty, \quad (2)$$

$$\sigma_{\mathrm{ex}} \vec{E}_{\mathrm{ex}} \hat{n} = \sigma_{\mathrm{f}} \vec{E}_{\mathrm{f}} \hat{n}, r \to r_{\mathrm{ex}} \quad (3)$$

$$\sigma_{\mathrm{f}} \vec{E}_{\mathrm{f}} \hat{n} = \sigma_{\mathrm{in}} \vec{E}_{\mathrm{in}} \hat{n}, r \to r_{\mathrm{in}} \quad (4)$$

$$E_{\mathrm{ex}} \hat{t} = E_{\mathrm{f}} \hat{t}, r \to r_{\mathrm{ex}} \quad (5)$$

$$E_{\mathrm{f}} \hat{t} = E_{\mathrm{in}} \hat{t}, r \to r_{\mathrm{in}} \quad (6)$$

The boundary conditions for the electric field components $\hat{n}$ (normal) and $\hat{t}$ (tangential) follow from $\nabla \times \vec{E} = 0$ and no net surface charge.

To calculate the electric stress acting on the capsule interface, we need to calculate the Maxwell electric stress tensors. For a dielectric material it is given by: $T_{k,i,j} \equiv \varepsilon_0 \varepsilon (E_i E_j - \frac{1}{2}\delta_{ij} E^2)$. Here, ε_0 is the vacuum permittivity, the indices i and j refer to the coordinates r, θ and φ, while the subscript k indicates the electric field on the exterior fluid side (k = 1) and the film side (k = 2) of the exterior interface. In this case, only the rr and $r\theta$ components of the Maxwell stress tensor are important ($\theta\theta$ and $\varphi\varphi$ components do not apply net force densities to the drop, while the other components are zero [8]). Here the rr electric stress tensor components for the exterior capsule interface are given by:

$$T_{1,rr}(r_{\mathrm{ex}}, \theta) = \frac{1}{2} \varepsilon_0 \varepsilon_{\mathrm{ex}} E_0^2 \left(\beta_{1,\mathrm{ex}}^2 \cos^2\theta - \gamma_{1,\mathrm{ex}}^2 \sin^2\theta\right), \quad (7)$$

$$T_{2,rr}(r_{\mathrm{ex}}, \theta) = \frac{1}{2} \varepsilon_0 \varepsilon_{\mathrm{f}} E_0^2 \left(\beta_{2,\mathrm{ex}}^2 \cos^2\theta - \gamma_{2,\mathrm{ex}}^2 \sin^2\theta\right). \quad (8)$$

The $r\theta$ stress tensor components for the exterior capsule interface are given by:

$$T_{1,r\theta}(r_{\mathrm{ex}}, \theta) = \varepsilon_0 \varepsilon_{\mathrm{ex}} E_0^2 \beta_{1,\mathrm{ex}} \gamma_{1,\mathrm{ex}} \cos\theta \sin\theta, \quad (9)$$

$$T_{2,r\theta}(r_{\mathrm{ex}}, \theta) = \varepsilon_0 \varepsilon_{\mathrm{f}} E_0^2 \beta_{2,\mathrm{ex}} \gamma_{2,\mathrm{ex}} \cos\theta \sin\theta. \quad (10)$$

The rr stress tensor components for the interior capsule interface are given by:

$$T_{2,rr}(r_{\mathrm{in}}, \theta) = \frac{1}{2} \varepsilon_0 \varepsilon_{\mathrm{f}} E_0^2 \left(\beta_{2,\mathrm{in}}^2 \cos^2\theta - \gamma_{2,\mathrm{in}}^2 \sin^2\theta\right), \quad (11)$$

$$T_{2,rr}(r_{\mathrm{in}}, \theta) = \frac{1}{2} \varepsilon_0 \varepsilon_{\mathrm{in}} E_0^2 (\beta_{3,\mathrm{in}}^2 \cos^2\theta - \gamma_{3,\mathrm{in}}^2 \sin^2\theta). \quad (12)$$

And lastly, the $r\theta$ stress tensor components for the interior capsule interface are given by:

$$T_{2,r\theta}(r_{\mathrm{in}}, \theta) = \varepsilon_0 \varepsilon_{\mathrm{f}} E_0^2 \beta_{2,\mathrm{in}} \gamma_{2,\mathrm{in}} \cos\theta \sin\theta, \quad (13)$$

$$T_{3,r\theta}(r_{\mathrm{in}}, \theta) = \varepsilon_0 \varepsilon_{\mathrm{in}} E_0^2 \beta_{3,\mathrm{in}} \gamma_{3,\mathrm{in}} \cos\theta \sin\theta. \quad (14)$$

The β and γ coefficients depend on the radii of the interfaces and the conductivities of the particle layer fluids:

$$\beta_{1,\mathrm{ex}} = \beta_{2,\mathrm{ex}} \frac{\sigma_{\mathrm{f}}}{\sigma_{\mathrm{ex}}} = \frac{3\sigma_{\mathrm{f}}[2r_{\mathrm{in}}^3(\sigma_{\mathrm{in}} - \sigma_{\mathrm{f}}) + r_{\mathrm{ex}}^3(2\sigma_{\mathrm{f}} + \sigma_{\mathrm{in}})]}{2r_{\mathrm{in}}^3(\sigma_{\mathrm{ex}} - \sigma_{\mathrm{f}})(\sigma_{\mathrm{f}} - \sigma_{\mathrm{in}}) + r_{\mathrm{ex}}^3(2\sigma_{\mathrm{ex}} + \sigma_{\mathrm{f}})(2\sigma_{\mathrm{f}} + \sigma_{\mathrm{in}})}, \quad (15)$$

$$\beta_{2,\mathrm{in}} = \beta_{3,\mathrm{in}} \frac{\sigma_{\mathrm{in}}}{\sigma_{\mathrm{f}}} = \frac{9r_{\mathrm{ex}}^3 \sigma_{\mathrm{ex}} \sigma_{\mathrm{in}}}{2r_{\mathrm{in}}^3(\sigma_{\mathrm{ex}} - \sigma_{\mathrm{f}})(\sigma_{\mathrm{f}} - \sigma_{\mathrm{in}}) + r_{\mathrm{ex}}^3(2\sigma_{\mathrm{ex}} + \sigma_{\mathrm{f}})(2\sigma_{\mathrm{f}} + \sigma_{\mathrm{in}})}, \quad (16)$$

$$\gamma_{1,\mathrm{ex}} = \gamma_{2,\mathrm{ex}} = -\frac{3\sigma_{\mathrm{ex}}[r_{\mathrm{in}}^3(\sigma_{\mathrm{f}} - \sigma_{\mathrm{in}}) + r_{\mathrm{ex}}^3(2\sigma_{\mathrm{f}} + \sigma_{\mathrm{in}})]}{2r_{\mathrm{in}}^3(\sigma_{\mathrm{ex}} - \sigma_{\mathrm{f}})(\sigma_{\mathrm{f}} - \sigma_{\mathrm{in}}) + r_{\mathrm{ex}}^3(2\sigma_{\mathrm{ex}} + \sigma_{\mathrm{f}})(2\sigma_{\mathrm{f}} + \sigma_{\mathrm{in}})} \quad (17)$$

$$\gamma_{2,\mathrm{in}} = \gamma_{3,\mathrm{in}} = -\beta_{3,\mathrm{in}}. \quad (18)$$

The total electric stress tensor components rr (normal to the capsule interface) and $r\theta$ (tangential to the capsule interface) are then calculated as:

$$\Delta T_{rr}(r_{\text{ex}}, r_{\text{in}}, \theta) = \left\{\left[T_{1,rr}(r_{\text{ex}}, \theta) - T_{2,rr}(r_{\text{ex}}, \theta)\right]^2 + [T_{2,rr}(r_{\text{in}}, \theta) - T_{3,rr}(r_{\text{in}}, \theta)]^2\right\}/r_{\text{f}}^2 \quad (19)$$

$$\Delta T_{r\theta}(r_{\text{ex}}, r_{\text{in}}, \theta) = \left\{\left[T_{1,r\theta}(r_{\text{ex}}, \theta) - T_{2,r\theta}(r_{\text{ex}}, \theta)\right]^2 + [T_{2,r\theta}(r_{\text{in}}, \theta) - T_{3,r\theta}(r_{\text{in}}, \theta)]^2\right\}/r_{\text{f}}^2, \quad (20)$$

where r_{f} is the mid-surface radius of the capsule, defined as: $r_{\text{f}} = r_{\text{ex}} - d/2 = r_{\text{in}} + d/2$. Because the electric stress components are non-uniform, they have to be balanced by non-uniform forces. For a particle-covered capsule, EHD-induced viscous forces can be neglected due to the incompressibility of the particles at the capsule interface. A capsule suspended in a fluid has two interfaces and two interfacial surface tensions (particle-drop and particle-surrounding fluid). Because the particle layer on the capsule is thin, we define surface tension γ as the average (mid-surface) of the two surface tensions. The non-uniform efficient surface tension is here defined as: $\gamma = \gamma_0 + \delta\gamma\cos 2\theta$, where γ_0 is a uniform surface tension and $\delta\gamma$ is a variable surface tension amplitude that is determined from the tangential stress balance (Equation 21). The normal electric stress component is balanced by the Laplace pressure, while the tangential component is balanced by the surface tension gradient:

$$\Delta T_{rr}(r_{\text{ex}}, r_{\text{in}}, \theta) = 2\gamma(\theta)H(s, \theta), \quad (21)$$

$$\Delta T_{r\theta}(r_{\text{ex}}, r_{\text{in}}, \theta) = \nabla\gamma\,, \quad (22)$$

where $H(s, \theta)$ is the mean curvature of the capsule [9]. The deformed capsule shapes can be described by: $r(\theta, \varphi) = r_{\text{f}}\hat{\boldsymbol{r}} + \boldsymbol{u}(\theta, \varphi)$, with the unit vector $\hat{\boldsymbol{r}}$ in the r direction and the displacement vector $\boldsymbol{u}$ parameterized as [10]: $u_{\text{r}} = s(3\cos^2\theta - 1)/2$ and $u_\theta = -s\sin\theta\cos\theta$, where s is an overall deformation amplitude. Here, we assume that the capsule deformations are small compared to the capsule radius r_{f} ($s \ll r_{\text{f}}$), and that $0 \le \theta \le \pi$. A series expansion to the first order yields $2H(s, \theta) = -(2r_{\text{f}} + s + 3s\cos 2\theta)/r_{\text{f}}^2$, $\lim_{s\to 0} 2H(s, \theta) \to -2/r_{\text{f}}$, which is the curvature of a non-deformed spherical capsule.

The capsule deformation is defined as: $D = (d_{||} - d_{\perp})/(d_{||} + d_{\perp})$, where $d_{||}$ and $d_{\perp}$ are the drop axes parallel and perpendicular to the electric field direction, respectively: $d_{||} = 2u_{\text{r}}(\theta = 0) = 2(r_{\text{f}} + s)$ and $d_{\perp} = 2u_r(\theta = \pi) = 2r_{\text{f}} - s$. The capsule deformation can then be expressed as:

$$D = \frac{3s}{4r_{\text{f}} + s} \approx \frac{3s}{4r_{\text{f}}}, \quad (23)$$

By solving Equations (21) and (22) for the deformation amplitude s, we get an expression for the capsule deformation. The resulting Pickering drop deformation to first order in film thickness d and second order in applied electric field strength E_0 is:

$$D = \frac{9E_0^2\varepsilon_0}{16\gamma_0\sigma_{\text{f}}(2\sigma_{\text{ex}} + \sigma_{\text{in}})^3}[\alpha_0 + \alpha_1 d + O(d^2)], \quad (24)$$

where the coefficients are:

$$\alpha_0 = r_{\text{f}}\sigma_{\text{f}}(2\sigma_{\text{ex}} + \sigma_{\text{in}})[\varepsilon_{\text{ex}}(\sigma_{\text{ex}} + \sigma_{\text{in}})^2 - 4\varepsilon_{\text{in}}\sigma_{\text{ex}}^2], \quad (25)$$

$$\begin{aligned}\alpha_1 = {} & \varepsilon_{\text{ex}}(\sigma_{\text{ex}} + \sigma_{\text{in}})[\sigma_{\text{in}}^2(\sigma_{\text{f}} - 2\sigma_{\text{ex}}) + \sigma_{\text{ex}}\sigma_{\text{f}}\sigma_{\text{in}} + 2\sigma_{\text{ex}}\sigma_{\text{f}}(\sigma_{\text{ex}} + 2\sigma_{\text{f}})] \\ & - 4\sigma_{\text{ex}}^2[\varepsilon_{\text{f}}(2\sigma_{\text{ex}} + \sigma_{\text{in}})(\sigma_{\text{f}} + \sigma_{\text{in}}) \\ & + \varepsilon_{\text{in}}(2\sigma_{\text{ex}}\sigma_{\text{f}} - 4\sigma_{\text{ex}}\sigma_{\text{in}} - 4\sigma_{\text{f}}^2 + 3\sigma_{\text{f}}\sigma_{\text{in}})].\end{aligned} \quad (26)$$

The capsule deformation can be reduced to the deformation of a pure drop (without a particle layer) where the tangential electric stress component is absorbed by the interface (electrohydrodynamic circulation flows are neglected) by $d \to 0$:

$$\lim_{d\to 0} D = \frac{9E_0^2 r_{\text{f}}\varepsilon_0[\varepsilon_{\text{ex}}(\sigma_{\text{ex}} + \sigma_{\text{in}})^2 - 4\varepsilon_{\text{in}}\sigma_{\text{ex}}^2]}{16\gamma_0(2\sigma_{\text{ex}} + \sigma_{\text{in}})^2} \quad (27)$$

We obtained the same drop deformation when we independently repeated these calculations for a drop without a particle layer (a drop with one radius and one interface). This expression is different

than the one found by Taylor [1], since we here balanced the tangential electric stress with a surface tension gradient instead of EHD-induced viscous stress. If we let the conductivity of the surrounding fluid go to zero, the deformation is reduced to the special case of a conducting drop surrounded by a perfect insulator [11]:

$$\lim_{\sigma_{ex}\to 0} D = \frac{9\varepsilon_{ex}\varepsilon_0 E_0^2 r_f}{16\gamma_0}. \quad (28)$$

In our numerical calculations we have used the following parameters for:

A PE Pickering drop: $r_f = 1.22$ mm, $d = 50$ µm, $\sigma_{ex} = 50$ pSm^{-1}, $\sigma_{in} = 5$ pSm^{-1}, $\gamma_0 = 4.5$ mNm^{-1}, $\varepsilon_0 = 8.854 \times 10^{-12}$ Fm^{-1}, $\varepsilon_{ex} = 4.7$, $\varepsilon_f = 2.2$, $\varepsilon_{in} = 2.8$, $\mu_{in} = 0.05$ Pa · s, $\mu_{ex} = 0.75$ Pa · s.

A clay Pickering drop: $r_f = 1.22$ mm, $d = 5$ µm, $\sigma_{ex} = 50$ pSm^{-1}, $\sigma_{in} = 5$ pSm^{-1}, $\gamma_0 = 4.5$ mNm^{-1}, $\varepsilon_0 = 8.854 \times 10^{-12}$ Fm^{-1}, $\varepsilon_{ex} = 4.7$, $\varepsilon_f = 6$, $\varepsilon_{in} = 2.8$, $\mu_{in} = 0.05$ Pa · s, $\mu_{ex} = 0.75$ Pa · s.

Drops in Alternating Current (AC) Fields

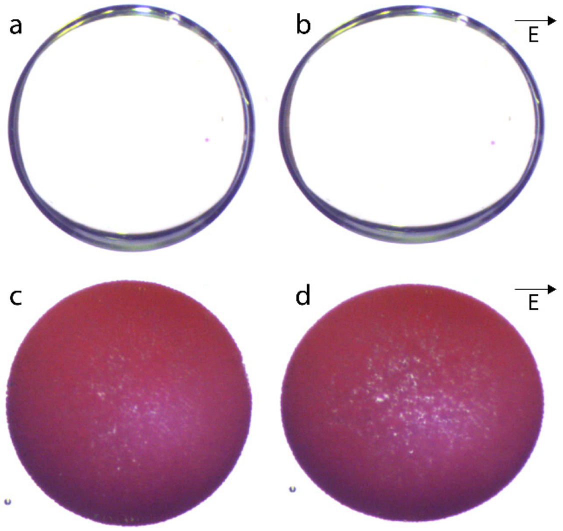

Figure S3. Deformation of a particle-free silicone oil drop and a PE Pickering drop subjected to an AC electric field. A particle-free silicone oil drop and a Pickering drop covered by 50 µm PE particles suspended in castor oil before (**a, c**) and after (**b, d**) the application of a 100 Hz electric field of strength 550 V/mm. The drop radii are 1.3 mm. The measured steady-state deformations are approximately 0.047 and 0.055 for the Pickering drop and silicone oil drop, respectively.

Particle layers at drop interfaces can provide resistance to deformation if the particles are jammed (if the particle packing is sufficiently high for particle motion to be prohibited). The particle layers on the PE Pickering drops in this paper are prepared such that the particle layer is not jammed (the particle packing is around 0.84 ± 0.02, just below the maximal theoretical packing for spheres at a flat interface, which is 0.91). Deformation experiments were performed on a pure silicone oil drop (radius 1.3 mm) and a PE-covered Pickering drop (radius 1.3 mm) when subjected to an AC electric field with a strength of 550 V/mm and a frequency of 100 Hz (Supplementary Figure S3). The field strength of 550 V/mm was chosen to produce large deformations (the maximum field strength provided by our setup), while we decided to use a frequency of 100 Hz to avoid charge build-up at the drop interfaces.

We found that the deformation of PE Pickering drops (≈ 0.047) was slightly smaller than that of the silicone oil drops (≈ 0.055) and, unlike similar drops in DC electric fields, both drops deform into a prolate geometry. Because the period of the frequency of the electric field is much higher than the inverse of the Maxwell-Wagner charge build-up time of the drops (around 1 s for this system), charges do not have time to accumulate at the drop interfaces. As a result, the drops behave as

dielectrics that polarize and are stretched by the electric field (into a prolate shape). Because the particle monolayer is thin (50 μm), polarization of the particle layer does not contribute significantly to the Pickering drop deformation in the AC electric field (100 Hz), and the deformation should be similar to the deformation of the pure silicone oil drop. The results show that resistance to drop deformation is not mainly caused by particle layer elasticity or by a change in Laplace pressure.

References

1. Taylor, G. Studies in electrohydrodynamics. I. The circulation produced in a drop by electrical field. *Proc. R. Soc. A Math. Phys. Eng. Sci.* **1966**, *291*, 159–166.
2. Melcher, J.R.; Taylor, G.I. Electrohydrodynamic—A review of role of interfacial shear stresses. *Annu Rev Fluid Mech.* **1969**, *1*, 111–146.
3. Saville, D.A. Electrohydrodynamics: The taylor-melcher leaky dielectric model. *Annu. Rev. Fluid Mech.* **1997**, *29*, 27–64.
4. Vizika, O.; Saville, D. The electrohydrodynamic deformation of drops suspended in liquids in steady and oscillatory electric fields. *J. Fluid Mech.* **1992**, *239*, 1–21.
5. Torza, S.; Cox, R.; Mason, S. Electrohydrodynamic deformation and burst of liquid drops. *Philos. Trans. R. Soc. Lond. A Math. Phys. Eng. Sci.* **1971**, *269*, 295–319.
6. Ouriemi, M.; Vlahovska, P.M. Electrohydrodynamic deformation and rotation of a particle-coated drop. *Langmuir* **2015**, *31*, 6298–6305.
7. Mikkelsen, A.; Dommersnes, P.; Fossum, J.O. Electric stress-induced slip lines in jammed particle monolayers. *Rev. Cub. Fis.* **2016**, *33*.
8. Yamamoto, T.; Aranda-Espinoza, S.; Dimova, R.; Lipowsky, R. Stability of spherical vesicles in electric fields. *Langmuir* **2010**, *26*, 12390–12407.
9. Russell, L.K. Mean curvature of a deformed spherical surface. *AIAA J.* **1971**, *9*, 1882–1883.
10. Winterhalter, M.; Helfrich, W. Deformation of spherical vesicles by electric-fields. *J. Colloid Interface Sci.* **1988**, *122*, 583–586.
11. Allan, R.; Mason, S. Particle behaviour in shear and electric fields. I. Deformation and burst of fluid drops. *Proc. R. Soc. Lond. A Math. Phys. Eng. Sci.* **1962**, *267*, 45–61.

© 2017 by the authors. Submitted for possible open access publication under the terms and conditions of the Creative Commons Attribution (CC BY) license (http://creativecommons.org/licenses/by/4.0/).
